# Polo-like kinase 1 promotes Cdc42-induced actin polymerization for asymmetric division in oocytes

**DOI:** 10.1098/rsob.220326

**Published:** 2023-03-08

**Authors:** Wai Shan Yuen, Qing Hua Zhang, Anne Bourdais, Deepak Adhikari, Guillaume Halet, John Carroll

**Affiliations:** ^1^ Department of Anatomy and Developmental Biology and Development and Stem Cells Program, Monash Biomedicine Discovery Institute, Monash University, Clayton, Victoria 3800, Australia; ^2^ University of Rennes, CNRS, IGDR - UMR 6290, F-35000 Rennes, France

**Keywords:** Plk1, Cdc42, actin, meiosis, oocyte, polarity

## Abstract

Polo-like kinase I (Plk1) is a highly conserved seronine/threonine kinase essential in meiosis and mitosis for spindle formation and cytokinesis. Here, through temporal application of Plk1 inhibitors, we identify a new role for Plk1 in the establishment of cortical polarity essential for highly asymmetric cell divisions of oocyte meiosis. Application of Plk1 inhibitors in late metaphase I abolishes pPlk1 from spindle poles and prevents the induction of actin polymerization at the cortex through inhibition of local recruitment of Cdc42 and Neuronal Wiskott-Aldrich Syndrome protein (N-WASP). By contrast, an already established polar actin cortex is insensitive to Plk1 inhibitors, but if the polar cortex is first depolymerized, Plk1 inhibitors completely prevent its restoration. Thus, Plk1 is essential for establishment but not maintenance of cortical actin polarity. These findings indicate that Plk1 regulates recruitment of Cdc42 and N-Wasp to coordinate cortical polarity and asymmetric cell division.

## Graphical abstract

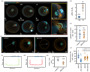

## 1. Introduction

Asymmetric cell division underlies essential developmental processes in all eukaryotes [1]. An extreme form of this occurs during the meiotic divisions of the oocyte. The mammalian oocyte undergoes two consecutive cell divisions to form a haploid oocyte and two small polar bodies [2]. These extremely asymmetric divisions allow the oocyte to retain cytoplasmic components essential for fertilization and early embryo development [3,4]. In mammalian oocytes, asymmetry is coupled to the migration of the centrally placed meiosis I spindle along its long axis towards the nearest cortex. This spindle migration is mediated via a cytoplasmic actin network formed by the activities of Formin and Spire type actin nucleators [5–11]. Immediately following the first meiotic division, the asymmetrically positioned maternal chromatin establishes the second meiotic spindle, which is maintained by cytoplasmic streaming beneath the cortex throughout arrest at metaphase II [12].

A critical event required for asymmetric division in the oocyte is the establishment of cortical polarity [3,4,13]. Cortical polarization results in the formation of an F-actin domain proximal to the spindle during late metaphase I and in metaphase II arrested oocytes [4,14]. The differentiation of the polar cortex in metaphase of both meiotic divisions contrasts with mitotic cell divisions where the cortical recruitment and activation of Rho and associated actin polymerization only occurs after anaphase when the central spindle forms [15]. The significance of the apparent difference in timing of cortical polarity is not clear but may reflect the stop-start nature of meiosis or the close association of the spindle with the cortex.

It has been long known that establishment of this oocyte polarity is induced by the sub-cortical presence of the meiotic chromatin [3,4,16]. More recently it has become evident that chromatin localizes Rho family small GTPases, Rac and Cdc42, to the cortex. The differential roles of these proteins in polar body formation and how they coordinated is still being established but Rac-GTP appears to be important for spindle anchoring in the cortex [17], and Cdc42-GTP is necessary for actin cap formation and absolutely required for polar body formation [18–20]. Cdc42-GTP-mediated actin polymerization is stimulated via the recruitment of Neuronal Wiskott-Aldrich Syndrome protein (N-Wasp) [12,18,20–23], which in turn activates the Arp2/3 complex to nucleate new actin filaments thereby creating the ‘actin cap’ [12,24].

The spindle- and chromatin-associated signalling for initiating the polarization of Cdc42-GTP is not yet fully understood. One candidate is the chromatin-associated Ran-GTP gradient [7,16]. DNA-coated beads injected in close proximity to the cortex of MII oocytes induce actin nucleation and this can be inhibited by expression of dominant negative Ran [25]. Furthermore, in MII-stage oocytes, disrupting Ran-GTP activity prevents cortical localization of Cdc42-GTP, N-Wasp and Arp2/3 [12]. While these findings provide strong evidence for Ran-GTP, particularly in MII-stage oocytes, the actual picture is more complex. Injection of beads coated with Ran-GTP or Regulator of Chromatin Condensation 1 (RCC1), which is Ran's nucleotide exchange factor, was not sufficient to induce actin polymerization [25] and inhibition of Ran-GTP in meiosis I does not prevent polar body extrusion [7,16], indicating other factors may be necessary for induction of cortical polarization.

Polo-like kinase 1 (Plk1) is a highly conserved chromatin and spindle-associated kinase critical for a number of mitotic events including spindle formation and cytokinesis [26,27]. At cytokinesis in mitotic cells, Plk1 activity is necessary for localizing Rho-GTP to the equatorial cortex where it induces actin polymerization and actomyosin contractility [28–31]. Plk1 achieves this via phosphorylation of the centralspindlin component, MgcRacGAP1/Cyk4, creating docking sites for the Rho-GEF, ECT2, which then communicates to the equatorial cortex where it activates Rho-GTP [31,32]. In oocyte meiosis, Plk1 inhibition late in metaphase I inhibits polar body formation and recruitment of Rho-GTP [33,34]. The role of Plk1 at cytokinesis led us to ask the question whether Plk1 may also play a role in polarizing the oocyte cortex in meiosis through recruitment of Cdc42-GTP.

Similar to mitosis, Plk1 is localized in a punctate manner at oocyte spindle poles and kinetochores and then at the spindle midbody during anaphase and telophase [35–37]. As such, spindle-associated Plk1 is in a prime location to initiate polarization and integrate the processes of cortical polarity with polar body extrusion. Due to the highly coordinated nature of oocyte meiosis, the role of Plk1 in establishing oocyte polarity may be dissected through carefully controlled temporal application of small molecule inhibitors. Our data indicate that Plk1 activity is necessary for the initiation of oocyte polarity but not its maintenance.

## 2. Results

### 2.1. Plk1 localization is consistent with a role in cortical polarization

**Figure 1.**
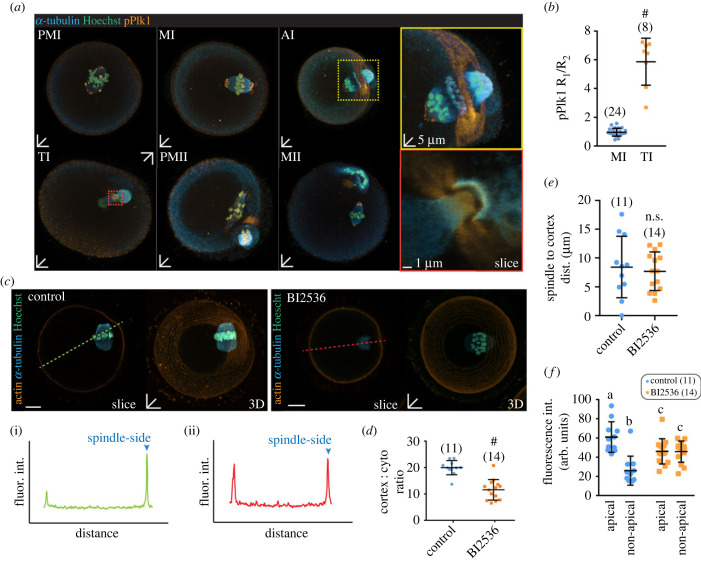
Localization of Plk1 and its effect on actin cap formation in meiosis I (*a*) pPlk1 localization in oocytes during various meiotic stages, pro-metaphase I (PMI), metaphase I (MI), anaphase I (AI) telophase I (TI), pro-metaphase II (PMII) and metaphase II (MII). Oocytes were stained for pPlk1 (orange), α-tubulin (blue) and DNA (teal). Novel atypical localization of pPlk1 highlighted as coloured insets of MI (green box), AI (yellow box) and TI (red box). (*b*) Ratio of pPlk1 fluorescence at apical (R1) versus non-apical (R2) cortex in TI versus MI oocytes. (*c*) Cortical actin levels in single-slice and three-dimensional stacks of control and BI2536-treated oocytes at 8 h post IBMX release stained for actin (orange), α-tubulin (cyan) and DNA (teal). Green and red dotted lines represent the linescan profiles shown in (i) control and (ii) BI2536, respectively. Arrowheads denote cortical actin fluorescence intensities nearest to the spindle pole. (*d*) Ratio of peak actin fluorescence at cortex to the average cytoplasm fluorescence in control and BI2536-treated oocytes. (*e*) Distance of the closest spindle pole to cortex distance in controls and BI2536-treated oocytes. (*f*) Peak apical and non-apical actin fluorescence intensity obtained from linescans of control and BI2536-treated oocytes. Black lines indicate mean ± s.d. *p < 0.05, ^#^*p* < 0.0001; different letters denote statistical significance. The total number of oocytes examined is in parentheses. Scale bars represent 10 µm unless otherwise stated.

Phosphorylated Plk1 (Thr210; pPlk1) immunofluorescence was performed on oocytes in different stages of meiosis from pro-metaphase I to metaphase II illustrates a punctate distribution on the spindle poles as well as on the centromeric region in both PMI, MI, PMII and MII oocytes (figure 1*a*). This result demonstrates active Plk1 is localized on the acentriolar microtubule organizing centres (aMTOCs) and is in broad agreement with previous findings [35–38]. We observed that upon anaphase I (AI), pPlk1 immunofluorescence shifts from aMTOCs to apical membrane surrounding the PB extrusion site (figure 1*a*, yellow box). At telophase I, pPlk1 immunofluorescence gathers at the midbody (figure 1*a*, red box) to a level several fold higher than that seen in metaphase I stage oocytes (figure 1*b*).

The punctate globular nature of pPlk1 immunofluorescence at spindle poles (figure 1*a*; electronic supplementary material, figure S1) is reminiscent of the liquid-like meiotic spindle domain (LISD) identified by So *et al.* [38]. We tested this possibility but confirmed there was no co-localization between pPlk1 and the LISD resident, TACC3 (electronic supplementary material, figure S1A). Further, the Plk1 inhibitor Volasertib caused the loss of pPlk1 from spindle poles but had no effect on TACC3 localization (electronic supplementary material, figure S1B,D,E). Conversely, disrupting LISDs using the Aurora A inhibitor, Alisertib, caused the loss of TACC3 but had no effect on pPlk1 (electronic supplementary material, figure S1C–E). Thus, consistent with So *et al*., Plk1 does not appear to be integral to LISDs in mouse oocytes.

### 2.2. Plk1 inhibition prevents cortical actin accumulation in meiosis I

We resolved to test the role of Plk1 in the establishment of cortical polarity through application of a small molecule inhibitor, BI2536, at 8 h post IBMX release, which is defined here as late metaphase I. This time point was chosen based on preliminary observations that showed in most oocytes the spindle had migrated to the cortex and that cortical polarity had not been established. Due to the acute nature of polarization and relatively small surface area to volume ratio of the oocyte, we used a brief treatment of BI2536 at concentration of 5 µM for most experiments. To test for specificity, we replicated the experiments using a concentration of 250 nM and found the same effect (electronic supplementary material, figure S2C). Oocytes were fixed 1 h after application of BI2536 and stained for actin. Linescan profiles show that control oocytes have a well-defined actin cap over the spindle pole relative to the opposite cortex (figure 1*c*(i)). This was calculated as a ratio of the peak fluorescence and the mean cytoplasm fluorescence as depicted in the electronic supplementary material, figure S1A. However, in BI2536-treated oocytes, no obvious polarized actin cap was detected, and actin was evenly distributed around the cortex (figure 1c(ii)). The cortex : cytoplasm ratio, confirms Plk1 inhibition causes a significant decrease in levels of actin over the spindle (figure 1*d*). This was calculated of the peak apical fluorescence as a ratio of the mean cytoplasmic fluorescence intensity (electronic supplementary material, figure S2A). This decrease in polarized actin in these oocytes was not due to a greater distance of the spindle from the cortex (figure 1*e*). Further analysis of the actin fluorescence intensity values confirmed the difference in apical and non-apical actin levels was effectively ablated by BI2536 treatment (figure 1*f*). Interestingly, this analysis also revealed that while BI2536-treated oocytes showed no difference between apical and non-apical actin, the overall cortical actin was increased above non-apical levels in control oocytes (figure 1*f*). We attribute this baseline increase to the release of actin monomers from the apical cortex leading to an increase in F-actin throughout the cortex.

**Figure 2.**
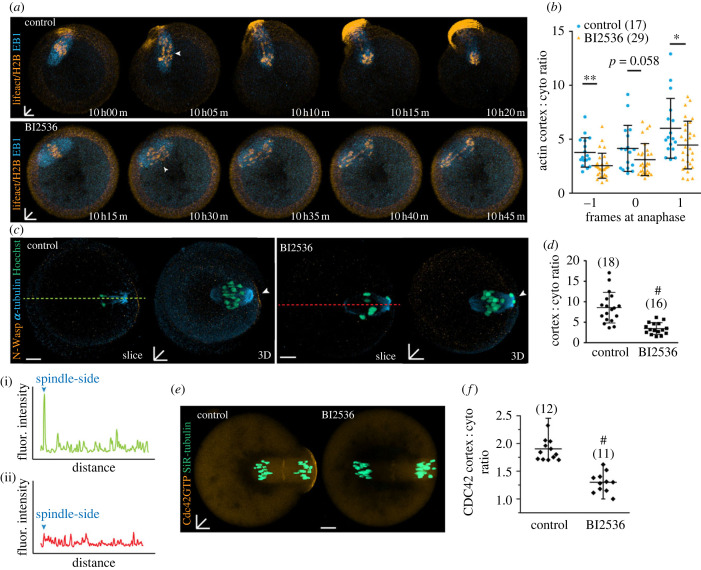
Plk1 inhibition affects cortical actin and N-Wasp localization during meiosis I. (*a*) Live-cell imaging of control and BI2536-treated oocytes microinjected with lifeact (orange), H2B (orange) and EB1 (blue) undergoing meiosis I. White arrowheads denote anaphase. Time stamps (hh : mm) are relative to IBMX release. (*b*) Ratio of peak cortical actin fluorescence to the average cytoplasm fluorescence in oocytes comparing the frame before (−1), during (0) and after (+1) anaphase (5 min intervals). (*c*) N-Wasp (orange) localization in control and BI2536-treated MI oocytes at 8 h post IBMX release in both single-slice and three-dimensional stacks stained for α-tubulin (cyan) and DNA (teal). White arrowheads denote expected position of cortical N-Wasp fluorescence. Green and red dotted lines represent the linescan profiles shown in (i) control and (ii) BI2536, respectively. (*d*) Ratio of peak N-Wasp fluorescence at cortex to the average cytoplasm fluorescence in control and BI2536-treated oocytes. (*e*) Live-cell images of control and BI2536-treated oocytes microinjected with Cdc42-GTP biosensor EGFP-wGBD [18] (orange) and stained with SiR-DNA (teal) at 9.5 post milrinone release. Chromosomes were labelled with Sir-DNA. Images are compressed Z-stacks of 15–20 consecutive confocal frames spanning the spindle volume. (*f*) Ratio of peak Cdc42-GTP fluorescence at cortex to the average cytoplasm fluorescence in control and BI2536-treated oocytes. Black lines indicate mean ± s.d. ^#^*p* < 0.0001, **p < 0.01, *p < 0.05. The total number of oocytes examined is in parentheses. Scale bars represent 10 µm.

Next, we performed live-cell imaging to investigate the kinetics of actin cap formation and the effect of Plk1 inhibition on meiotic progression. Oocytes were injected with Lifeact to monitor actin filaments, H2B-mcherry to monitor chromatin and EB1-GFP to visualize microtubules. Oocytes were imaged in BI2536-containing or control media starting 8 h after release from IBMX. In control oocytes, the actin cap appears when the first meiotic spindle approaches the cortex and increases in fluorescence following anaphase, and ultimately remains constrained to the membrane destined to form the first polar body (figure 2*a*). We found that BI2536 treatment did not affect the timing of anaphase onset; however, no obvious actin cap was formed prior or subsequent to anaphase and the oocyte was unable to extrude the first polar body (figure 2*a*). The effect of BI2536 was quantified by measuring the cortical-cytoplasmic ratio of actin fluorescence at anaphase, 5 min prior to anaphase onset and 5 min after. Consistent with the phalloidin data (figure 1*e*), the ratio was consistently lower in BI2536-treated oocytes at each time point analysed (figure 2*b*). Together, these data indicate a previously unidentified role for Plk1 in establishing the polarized actin cortex.

### 2.3. Plk1 inhibition reduces N-Wasp and Cdc42 polarization in meiosis I

We asked whether BI2536 inhibits the recruitment of N-Wasp and Cdc42-GTP to the cortex overlying the spindle. Oocytes were treated with BI2536 as described above and examined for N-Wasp localization using immunofluorescence. An N-Wasp cap was consistently observed in control oocytes (figure 2*c*(i)), whereas in BI2536-treated oocytes, no evidence for polarized N-Wasp could be observed (figure 2*c*(ii)). Quantification using linescan ratios confirmed inhibition of Plk1 leads to a loss of N-Wasp labelling in the oocyte cap region (figure 2*d*).

N-Wasp is recruited to the cortex by Cdc42, which is the most upstream regulator of actin polymerization. We have previously demonstrated Cdc42-GTP is essential for the polarized actin cortex [18] and oocyte-specific depletion of Cdc42 prevents actin cap formation and polar body formation [19]. Thus, we next set out to establish if Plk1 is necessary for polarized localization of Cdc42-GTP. Oocytes were injected with the Cdc42-GTP probe, eGFP-WGBD, and treated with BI2536 in late metaphase I. BI2536 treatment abolished any detectable Cdc42-GTP over the region of the spindle while control oocytes reliably demonstrated polarized Cdc42-GTP (figure 2*e,f*). These data indicate that Plk1 is responsible for the recruitment of active Cdc42, which in turn recruits N-Wasp, ultimately leading to actin polymerization through the Arp2/3 pathway.

### 2.4. Plk1 inhibition reduces spindle asymmetry of tyrosinated α-tubulin

A previous study demonstrated that Cdc42 activity in the cortex overlying the spindle is responsible for an accumulation of tyrosinated α-tubulin in the proximal end of the spindle [39]. Thus, we hypothesized that if Plk1 specifically prevents Cdc42 activation at the overlying spindle cortex, it should also inhibit polarization of the asymmetric accumulation of tyrosinated α-tubulin.

**Figure 3.**
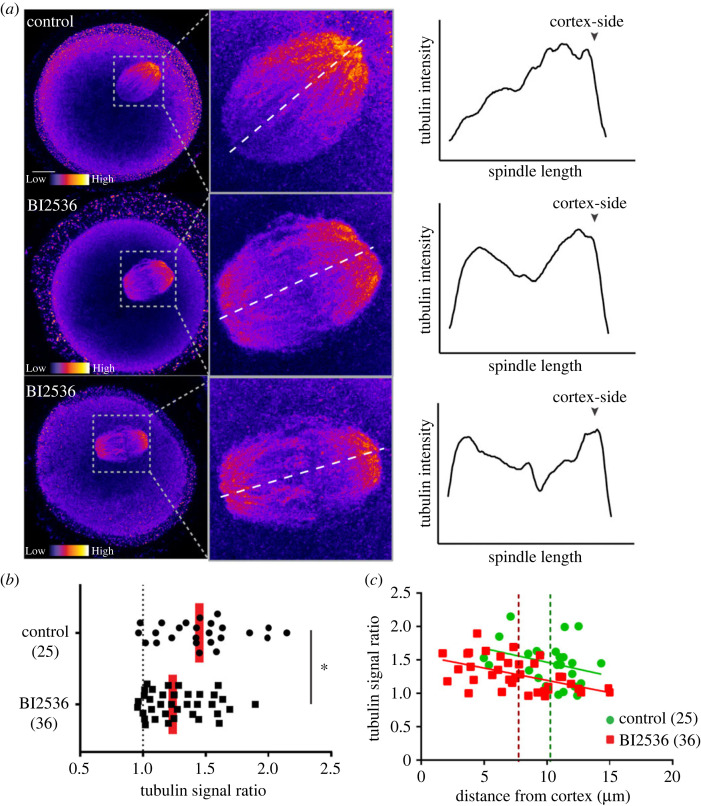
Plk1 reduces localization of apical tyrosinated α-tubulin on the MI spindle (*a*) Representative images of control and BI2536-treated oocytes stained for tyrosinated α-tubulin (red-heatmap; warmer colours indicate higher fluorescence intensity) at 9 h post IBMX release. Images are sum intensity *z*-axis projections showing the whole oocyte (left) and a magnified view of the spindle (right). Graphs are linescans of tyrosinated α-tubulin intensity across the spindle as indicated by the white dotted line shown on the magnified inset. (*b*) Spindle asymmetry was quantified as the ratio of the cortical half to the oocyte half. Each dot represents a single spindle. Red line, median *p < 0.05. (*c*) Tyrosinated α-tubulin signal ratio of every control and BI2536-treated oocyte plotted as a function of its spindle distance to the cortex. Each dot represents a single oocyte. Control (green) and BI2536 (red) mean spindle to cortex distance is represented by dotted lines.

To test this hypothesis, MI oocytes were treated with BI2536 at 7.5 h after IBMX washout, released for 1.5 h and stained for tyrosinated α-tubulin. As previously described [39], control oocytes showed strong asymmetry of tyrosinated α-tubulin biased towards the cortex-facing spindle pole (figure 3*a*). By contrast, BI2536-treated oocytes showed reduced spindle asymmetry of tyrosinated α-tubulin (figure 3*a,b*). We confirmed by plotting the asymmetry ratio against distance of spindle from the cortex that the ratio tended to be greater the closer spindle was to the cortex (figure 3*c*). Importantly, BI2536 has no effect on spindle distance from the cortex, confirming its effects are the result of inhibition of spindle-associated Plk1 rather than via impacting spindle positioning (figure 3*c*). These findings support the idea that spindle-associated Plk1 leads to activation of Cdc42 which drives two independent events, actin polymerization and tyrosinated α-tubulin distribution in the spindle.

### 2.5. Maintenance of cortical polarization in metaphase II is not regulated by Plk1

**Figure 4.**
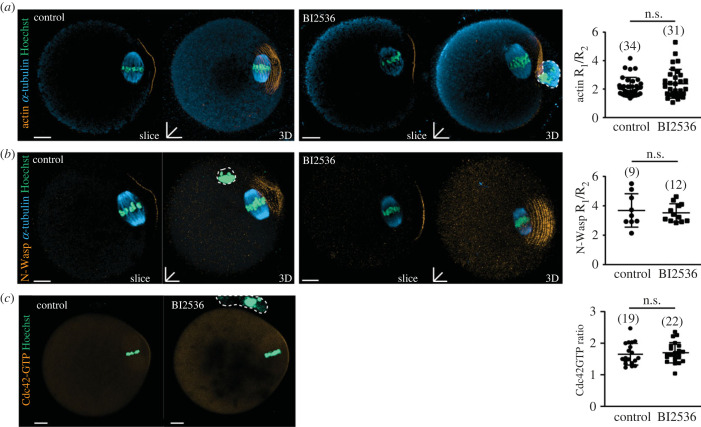
Cortical localization of actin, N-Wasp and Cdc42 are unaffected by Plk1 inhibition in MII oocytes. (*a*) Single-slice and three-dimensional stacks of superovulated control or BI2536-treated MII oocytes stained for actin (orange), α-tubulin (cyan) and DNA (teal). The respective R_1_/R_2_ ratio measured is to the right of the corresponding images. White dotted lines denote position of the first polar body if present. (*b*) Single-slice and three-dimensional stacks of superovulated control or BI2536-treated MII oocytes stained for N-Wasp (orange), α-tubulin (cyan) and DNA (teal). The respective R_1_/R_2_ ratio measured is to the right of the corresponding images. (*c*) Superovulated control or BI2536-treated MII oocytes microinjected with Cdc42-GTP biosensor (orange). Chromosomes were labelled with Sir-DNA. The respective R_1_/R_2_ ratio measured is to the right of the corresponding images. Scale bars represent 10 µm. Black lines indicate mean ± s.d. Dotted lines represent first polar body in the various z-projected images. *n.s*., non-significant, *p* > 0.05. The total number of oocytes examined is in parentheses. White dotted lines denote position of the first polar body if present.

To date, all experiments have focused on the role of Plk1 in establishing cortical polarity in meiosis I. To ask if Plk1 is involved in maintaining cortical polarity in MII-stage oocytes, we have examined the ability of BI2536 to dissipate the stable actin cortex that overlies the MII spindle. Ovulated MII-stage oocytes were treated with BI2536 for 2 h and actin examined using phalloidin staining. Remarkably, despite the dramatic effects seen in establishing polarity in MI, BI2536 treatment had no effect on actin distribution in MII-stage oocytes (figure 4*a*). This was calculated as a ratio of the fluorescence intensity of the apical and the non-apical cortex shown in the electronic supplementary material, figure S2B.

The lack of any effect on polarized actin MII-stage oocytes may be due to the inability to delocalize Cdc42 and N-Wasp or that polymerized actin is sufficiently stable so as not to be impacted by the loss of polarized Cdc42 and N-Wasp. To address this question, MII oocytes were treated with BI2536 and the distribution of the actin regulators was examined. The data clearly show that BI2536 has no effect on polarized distribution of N-Wasp (figure 4*b*) or Cdc42-GTP, (figure 4*c*) indicating that maintenance of the actin polymerization pathway in MII-stage oocytes is independent of Plk1. The lack of effect of N-Wasp recruitment during MII compared to MI was not due to differing protein levels (electronic supplementary material, figure S3A).

### 2.6. Actin and Plk1 collaborate in the control of polarized distribution of regulators of actin polymerization

**Figure 5.**
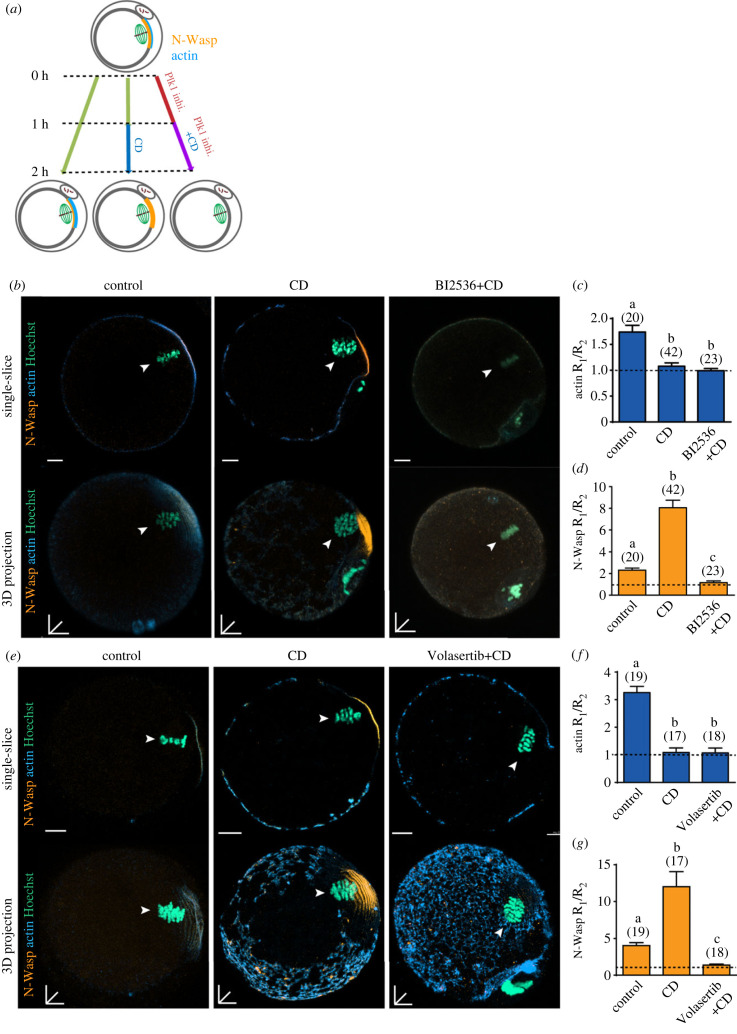
Plk1 inhibition abolished cortical N-Wasp polarization. (*a*) Schematic illustrating the experimental method performed. Superovulated MII oocytes were split into three groups; no drug control, CD-treated and CD plus BI2536/Volasertib. These groups were then fixed at the end of CD treatment. (*b*) Representative images of control MII, CD-treated, CD plus BI2536-treated groups. Oocytes were stained for N-Wasp (orange), actin (blue) and DNA (teal). White arrows denote MII chromatin. (*c*) Actin fluorescence ratio measured in MII oocytes from (*b*) without washout. (*d*) N-Wasp fluorescence ratio measured in MII oocytes from (*b*) without washout. (*e*) Representative images of control MII, CD-treated, CD plus Volasertib- treated groups. Oocytes were stained for N-Wasp (orange), actin (blue) and DNA (teal). White arrows denote expected position of polarized cortex. (*f*) Actin fluorescence ratio measured in MII oocytes from (*b*) without washout. (*g*) N-Wasp fluorescence ratio measured in MII oocytes from (*b*) without washout. Bar graphs indicate mean ± s.d. Different letters denote significant difference. The total number of oocytes examined is in parentheses. Scale bars represent 10 µm.

To further examine the mechanisms underlying establishing and maintaining cortical actin polarity, we next inhibited actin polymerization in MII oocytes using cytochalasin D (CD) and examined the effect of BI2536 on the distribution of polarized N-Wasp and actin. Ovulated MII oocytes were incubated for 1 h in control media followed by 1 h in CD in the presence or absence of BI2536, or a second Plk1 inhibitor, Volasertib. Controls were incubated for 2 h in control medium to confirm *in vitro* culture had no effect on actin or N-Wasp localization (figure 5*a*). As expected, a 1 h exposure to CD alone completely abolished polarized actin polymerization (figure 5*b,c,e,f*). This CD-induced loss of actin was accompanied by a three- to fourfold increase in N-Wasp recruitment to the cortex (figure 5*b,d,e,g*), indicating the presence of a negative feedback pathway between polarized F-actin and N-Wasp recruitment. This is further supported by confirming there is no difference in N-Wasp protein levels in CD-treated and control oocytes (electronic supplementary material, figure S3B).

We next asked if Plk1 was necessary for the increase in N-Wasp in CD-treated oocytes by co-treating oocytes with BI2536 or Volasertib and CD (figure 5*a*). Using both inhibitors, Plk1 inhibition completely abolished the CD-induced increase in N-Wasp recruitment to the cortex suggesting that Plk1 plays a critical role in the recruitment of actin nucleating factors to the cortex (figure 5*b,d,e,g*). This is also evident in oocytes treated with a lower dose of BI2536 (250 nM; electronic supplementary material, figure S2C).

### 2.7. Plk1 inhibition prevents re-establishment of the polarized actin cap

**Figure 6.**
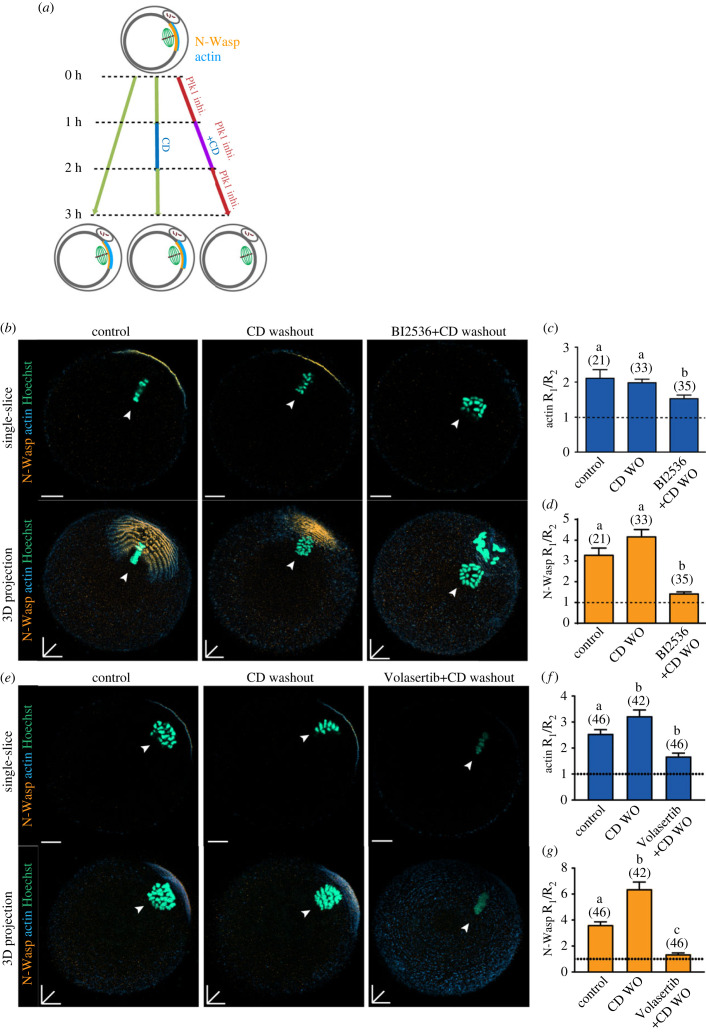
Plk1 is essential for cortical actin and N-Wasp establishment. (*a*) Schematic illustrating the experimental method performed. Superovulated MII oocytes were split into three groups; no drug control, CD-treated and CD plus BI2536/Volasertib. These groups were then washed out of CD and left to recover for an hour. (*b*) Representative images of control MII, CD-washout, CD washout plus BI2536-treated groups. Oocytes were stained for N-Wasp (orange), actin (blue) and DNA (teal). White arrows denote MII chromatin. (*c*) Actin fluorescence ratio measured in MII oocytes from (*b*) without washout. (*d*) N-Wasp fluorescence ratio measured in MII oocytes from (*b*) without washout. (*e*) Representative images of control MII, CD-washout, CD-washout plus Volasertib-treated groups. Oocytes were stained for N-Wasp (orange), actin (blue) and DNA (teal). White arrows denote expected position of polarized cortex. (*f*) Actin fluorescence ratio measured in MII oocytes from (*b*) without washout. (*g*) N-Wasp fluorescence ratio measured in MII oocytes from (*b*) without washout. Bar graphs indicate mean ± s.d. Different letters denote significant difference. The total number of oocytes examined is in parentheses. Scale bars represent 10 µm.

Next, we asked if inhibition of Plk1 prevents re-establishment of the actin cortex after CD washout (figure 6). In this scenario, as shown above, the cortex of CD-treated MII oocytes is ‘primed’ for recovery with high levels of N-Wasp, while the CD and BI2536-treated oocytes present with a cortical region bereft of N-Wasp, similar to MI oocytes prior to establishment of polarity. One hour after CD washout, MII oocytes recover polarized actin such that it is indistinguishable from controls (figure 6*b,c,e,f*). By contrast, in BI2536- and Volasertib-treated oocytes, washing out CD fails to fully support the restoration of polarity of actin or N-Wasp (figure 6*b,d,e,g*). Thus, as in MI stage oocytes, establishing polarity in the cortex of a CD-treated MII oocyte also requires active Plk1.

## 3. Discussion

Extreme asymmetric division in oocytes is essential for completion of the meiotic divisions and is the foundation for normal embryo development [40,41]. Achieving these meiotic divisions requires the establishment of a polarized actin cortex which demarcates the boundary of the future polar body. Our findings provide evidence for a new role of Plk1 activity in meiotic divisions—the establishment of cortical polarity.

Here we show that inhibition of Plk1 activity late in MI is sufficient to prevent the establishment of a polarized actin cortex in MI oocytes. This role for Plk1 was also revealed in MII-stage oocytes by first depolymerizing the actin cortex and allowing it to recover in the presence or absence of two different Plk1 inhibitors. Interestingly, in ovulated MII-stage oocytes in which there is a stable actin cap overlying the MII spindle, Plk1 activity is not necessary for its maintenance, thus we conclude that Plk1 is necessary for establishment but not maintenance of polarity in oocytes. In doing so we extend the many roles of Plk1 in mitosis [26,27] and meiosis [33,34] to include that of establishing oocyte polarity, thereby coordinating the highly asymmetric meiotic cell divisions.

The localization of Plk1 to spindle poles and kinetochores may in part explain the different patterns of cortical actin in MI and MII. In MI, actin remains restricted to the region immediately overlying the proximal spindle pole that approaches the cortex, while in MII, where the spindle lies parallel to the cortex, the actin cortex is broad, encompassing the entire region overlying the spindle. This difference in cortical actin organization likely reflects the different spindle orientations determining signalling molecule proximity to the cortex. The localization of Plk1 to spindle poles and kinetochores is one molecule among others [38], that is consistent with establishing the different patterns of actin polymerization.

The mechanism by which Plk1 enables polar actin polymerization appears to be by promoting the localization of the Cdc42/N-Wasp/Arp2/3 signalling complex to the region overlying the spindle. A specific role for Plk1 in localization of this Cdc42-mediated actin polymerization pathway is further supported by our data showing that Plk1 inhibition also prevents the Cdc42-induced polar distribution of tyrosinated α-tubulin in the MI spindle [39]. Thus, the inhibition of Plk1 at late metaphase I prevents the two known roles of polar-localized Cdc42 in oocyte meiosis, actin polymerization and tyrosinated α-tubulin.

It remains to be determined how Plk1 interacts with Ran-GTP or if they act independently on different aspects of the actin pathway. The lack of significant effect of dominant negative or constitutively active Ran constructs on first polar body formation [7,16] suggests the Ran-GTP gradient is not necessary for establishing polarity at MI. However, at MII, inhibition of the Ran-GTP gradient does inhibit Cdc42/Arp2/3 localization in the cortex [12,18], as well as actin polymerization in response to DNA-coated beads [25]. The apparent effectiveness of Ran-GTP in MII oocytes, which have an established actin cap, may indicate it plays a role in maintenance, rather than establishing, the polar distribution of Cdc42-GTP. Interestingly, one potential interface for Plk1 and Ran may be via Plk1-mediated phosphorylation of RanBP1 [42]. Ran-GTP production in mitosis is regulated by RanBP1 which is thought to control RCC1 loading onto chromatin and form a Ran/RanBP1/RCC1 complex in the cytoplasm that restrains Ran-GTP production [43]. This has not yet been explored in oocyte meiosis and may be a fruitful area for future research.

**Figure 7.**
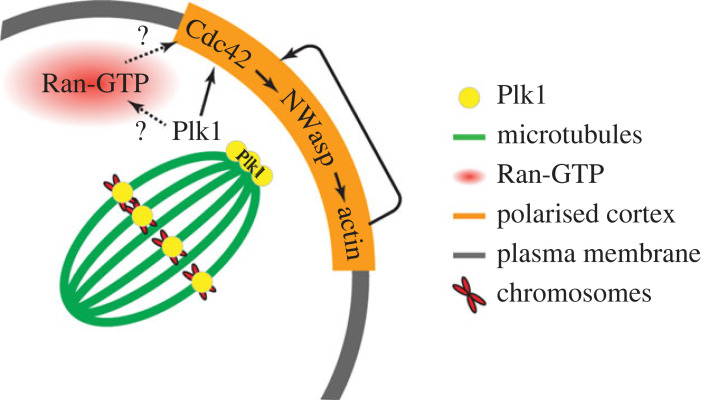
Schematic of hypothesized Plk1 regulation of cortical polarity establishment. Spindle-associated Plk1 is essential for the establishment of cortical polarity through the Cdc42 pathway. This is may be via Ran-GTP or other unknown pathways. Polarized actin has a negative regulatory feedback loop limiting further accumulation of Cdc42/N-Wasp.

Our data also reveal that actin polymerization in the cortex is under tight regulatory controls. The fourfold increase in cortical N-Wasp seen when actin is depolymerized using CD suggests actin itself, or an actin-binding protein, provides negative feedback at the level of N-Wasp (or Cdc42) to limit the extent of actin polymerization (figure 7). In other systems involving actin-mediated virus or vesicle motility, the interaction of N-Wasp with Arp2/3 or actin filaments accelerates N-Wasp exchange [44,45]. A similar actin/Arp2/3-mediated acceleration in turnover may explain the increased N-Wasp seen in the oocyte cortex when actin is depolymerized and points to a previously unappreciated highly dynamic feedback regulation of cortical actin (figure 7).

Close examination of our findings also reveals some evidence that Plk1-independent actin polymerization may be present in the oocyte cortex. In the presence of CD, we see a complete collapse of any actin polarity. On recovery after CD washout, the recovery of the polarized actin cortex is significantly reduced by the Plk1 inhibitors, but not completely abolished. The fact that Plk1 inhibitors prevented N-Wasp recovery to a greater extent than actin suggests that additional actin polymerization pathways may be present that can act redundantly with N-Wasp. In this context deletion of N-Wasp from oocytes does not abolish actin polymerization [46] and downregulation of other family members, WAVE and WASH, have been shown to suppress polar body formation [24,47,48]. More work is needed to know if the WAVE/WASH knock-down phenotype is caused by severe effects on spindle organization or via a direct effect on actin polymerization. Dissecting out the specific roles of the multitude of actin regulators in establishing and maintaining cortical polarity will require novel fast-acting specific small molecule inhibitors or new fast-acting targeted protein degradation approaches [49,50].

There is a precedent for spindle-associated Plk1 playing a role in influencing cortical behaviour. In HeLa cells, Plk1 activity on the spindle results in dissociation of dynein and dynactin from the cortex thereby stabilizing spindle positioning [51]. Our finding that Plk1 promotes localization of Cdc42 to the cortex reflects the role Plk1 plays at cytokinesis where it is necessary for localization of Rho at the cortex overlying the spindle midzone where it induces actomyosin contractility [28,30]. In meiosis, oocyte-specific deletion of Cdc42 does not impact chromosome dynamics at anaphase but causes a failure to form the actin cap and extrude the polar body [19]. This absolute requirement for Cdc42 for first polar body extrusion may involve the formation of actin filaments to support the contractile ring or it may also play a role in cortical softening [52] and/or membrane protrusion [20], all of which may be important early events for polar body extrusion. Our finding that spindle-associated Plk1 plays a critical role in recruiting Cdc42 to the overlying cortex identifies yet another role for this multi-functional protein in the events controlling cell division.

## 4. Experimental procedures

### 4.1. Oocyte collection and culture

The Animal Ethics Committee of Monash University approved all animal handling and experimental protocols, which were conducted in accordance with the Australian Code of Practice for the Care and Use of Animals for Scientific Purposes. C57BL/6J mice were obtained from Monash Animal Research Platform and housed under controlled environmental conditions with free access to water and food.

For *in vitro* maturation, GV oocytes were collected in M2 (Sigma) containing 200 µM 3-isobutyly-1-methylxanthine (IBMX). Culture was performed in drops of M16 medium (Sigma) under mineral oil (Sigma) at 37°C in a humidified atmosphere of 5% CO^2^ in air. For collection of MII-stage oocytes, mice were superovulated by sequential intraperitoneal injections of 10IU pregnant mare's serum gonadotropin (PMSG, Intervet) and 10IU human chorionic gonadotropin (hCG, Intervet) at timed intervals before oocyte collection. BI2536 (Sigma-Aldrich) and Volasertib (Selleckchem) were reconstituted in DMSO and used at 5 µM concentration in experiments. CD reconstituted in DMSO and used at 10 µM final concentration. All control groups were treated with DMSO as a vehicle control.

### 4.2. *In vitro* transcription and microinjections

cRNAs of Histone 2B-mCherry, Lifeact-mCherry, End Binding Protein 1-GFP mRNA, Cdc42-GTP probe EGFP-wGBD were prepared *in vitro* were transcribed using the SP6 or T7 mMessage mMachine Kit (Ambion) according to the manufacturer's instructions. mRNA (1.2 mg ml^−1^) was delivered using a microinjection apparatus consisting of Narishige micromanipulators mounted on a Zeiss S100TV inverted microscopy. A controlled delivery of approximately 5% oocyte volume was delivered to GV or MII-stage-arrested oocytes using a picopump. After injection, oocytes were washed out of IBMX and transferred in M16 medium.

### 4.3. Immunofluorescence and imaging

Oocytes or oocytes were fixed in a solution of 4% paraformaldehyde and 2% triton X-100 in PBS for 30 min at room temperature. Blocking was performed in PBS with 10% goat or donkey serum, 10% BSA and 2% Tween for 60 min at room temperature. Antibodies used for immuno-labelling: pPlk1 (1 : 200, Santa Cruz Biotechnology), N-Wasp (1 : 100, Cell Signaling Technology), TACC3 (AF5720-SP; R&D System), rat anti-tyrosinated α-tubulin (1 : 1000, Serotec, YL1/2) and FITC-conjugated mouse anti-α-tubulin (1 : 200, Alexa Fluor 488, Invitrogen) and detected with secondary antibody of Goat anti-Rabbit IgG antibody (1 : 1000, Alexa Fluor 555, Invitrogen). Phalloidin Alexa Fluor 555 (1 : 20, Cell Signaling Technology) was used where appropriate to stain actin. DNA was labelled using 10 min incubation in Hoechst 33 342 (10 µg ml^−1^, Sigma-Aldrich). Serial Z sections of fixed oocytes/oocytes were acquired at room temperature in a glass-bottomed dish using laser-scanning confocal microscope imaging system (SP8, Leica). For live-cell imaging, the microinjected oocytes were incubated in M2 media at 37°C under mineral oil and imaged with Leica SP8 confocal microscope. In some live imaging experiments, chromosomes were labelled with 1 µM Sir-DNA (Spirochrome)

### 4.4. Image and statistical analysis

Images were taken at equivalent settings within and across all experimental replicates. Data quantification was performed in FIJI using unscaled raw images/data. Mean membrane fluorescence intensity was measured using a threshold to isolate the membrane fluorescence then using FIJI to measure the average fluorescence intensity of each oocyte. Statistical analysis was performed using student's t-tests on data with numerical values, or Chi^2^ test on data with categorical values, with Sigma Plot or GraphPad Prism Software. Analysis was performed for at least three replicate experiments and represented as means and s.d., unless otherwise stated. Differences at *p* < 0.05 were considered significant. Level of significance is denoted by *p < 0.05, **p < 0.01, ***p < 0.001, *^#^p* < 0.0001. Columns with different letters are significantly different at least *p* < 0.05.

## Data Availability

Supplementary information is provided in the electronic supplementary material [53].
